# Asteroid Shower: Cutaneous Silica Granuloma with Asteroid Bodies

**DOI:** 10.3390/dermatopathology12010004

**Published:** 2025-01-31

**Authors:** Fadwa Ahmed, Christopher DiMarco

**Affiliations:** 1 Department of Dermatology, Warren Alpert Medical School, Brown University, Providence, RI 02903, USA; 2 Laboratory Medicine, Department of Pathology, Warren Alpert Medical School, Brown University, Providence, RI 02903, USA

**Keywords:** silica, foreign-body granuloma, cutaneous, sarcoid-like granuloma, asteroid bodies

## Abstract

Cutaneous silica granulomas are a form of foreign-body granulomatous reactions. They are characterized histopathologically by sarcoidal granulomas in association with silica crystals. Asteroid bodies, a classical histopathological feature of sarcoidosis, have not previously been reported in association with silica granulomas. Herein, we present the case of an 83-year-old man with an asymptomatic papule on the vertex scalp. Histopathology revealed a dermal granulomatous reaction to silica crystals. Asteroid bodies were observed in the cytoplasm of multinucleated giant cells. In the absence of systemic symptoms or laboratory findings suggestive of sarcoidosis, a final diagnosis of silica granuloma with asteroid bodies was made. While they have been observed in several other granulomatous reactions, the present case represents a novel association of asteroid bodies with silica granulomas.

## 1. Introduction

Cutaneous silica granulomas are a subset of foreign-body granulomatous reactions [1,2]. Although silica is a commonly occurring substance in the environment, few cases of cutaneous silica granulomas have been described. Clinically, they present as solitary or multiple papules or nodules months to years after inoculation of the skin with silica-containing materials, often through a traumatic or occupational exposure. History of previous trauma or scarring in the areas can potentially be clinical clues to this diagnosis. Histopathologically, cutaneous silica granulomas are characterized by mixed or sarcoid-like granulomatous infiltrates with multinucleated giant cells in association with irregular translucent crystalline structures of various sizes that are characteristically birefringent on polarized light microscopy. 

Despite the close histopathologic resemblance of cutaneous silica granulomas and cutaneous sarcoidal granulomas, asteroid bodies—a typical histopathological feature of sarcoidosis—have not previously been reported in silica granulomas. Herein, we describe a novel case of cutaneous silica granuloma associated with asteroid bodies. Physicians should be aware of the possibility of observing asteroid bodies in both sarcoidosis and foreign-body granulomatous reactions, including cutaneous silica granulomas. 

## 2. Case Report

**Figure 1:**
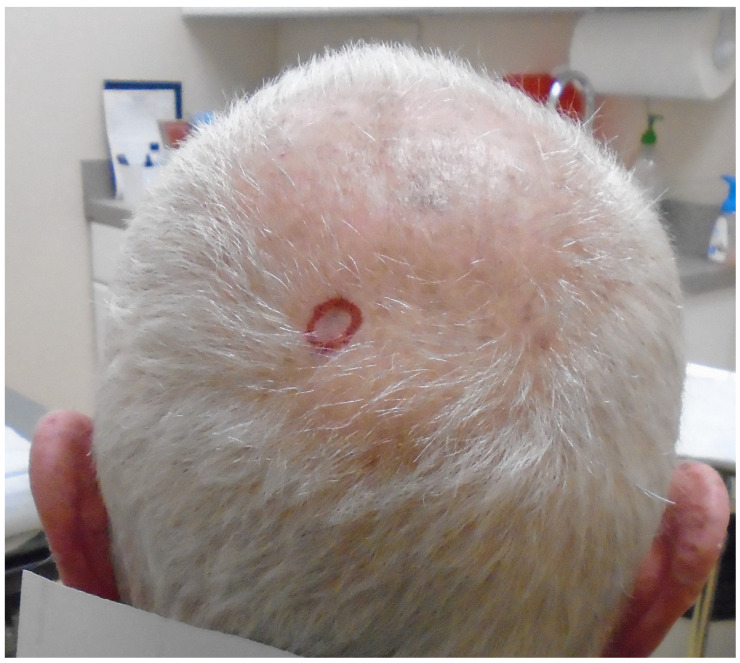
Clinical image of 8 mm depressed pinkish papule on the vertex scalp, circled in red.

An 83-year-old man with a history of cutaneous squamous cell carcinoma, prostate cancer, and hypothyroidism presented with an 8 mm depressed pinkish papule on the vertex scalp (Figure 1). Though he admitted to picking at the lesion, he denied any associated symptoms. Given clinical concern for squamous cell carcinoma in situ, a shave biopsy was obtained. 

**Figure 2:**
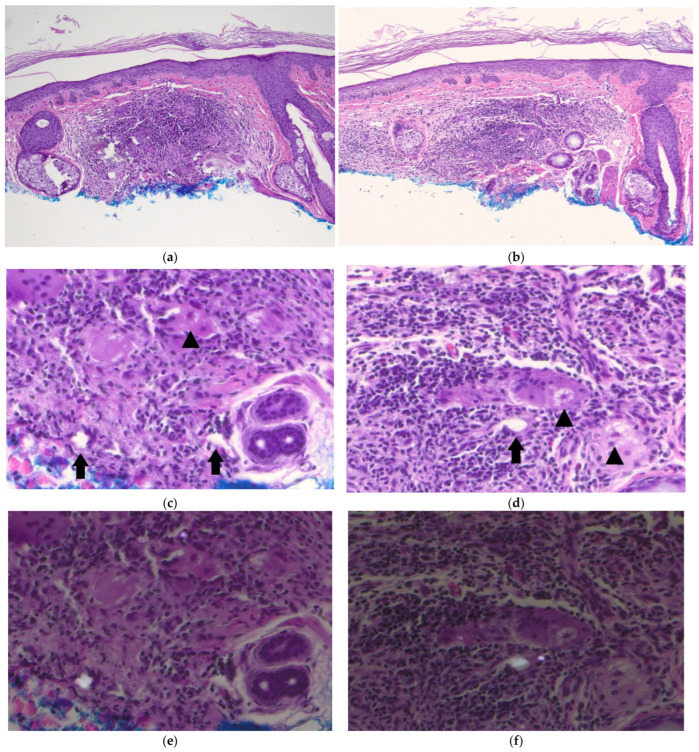
Silica granuloma with asteroid bodies: (**a**,**b**) low-power view showing dense granulomatous infiltrate in the dermis (H&E, ×100); (**c**,**d**) high-power image of the infiltrate, composed of lymphocytes, eosinophils, and histiocytes with foreign-body-type giant cells, highlighting representative anisotropic crystalline material (arrows) and asteroid bodies (arrowheads) found within the granulomatous inflammation (H&E, ×400); (**e**,**f**) partial polarization of (**c**,**d**) to show polarization of silica crystals (H&E, ×400).

Histologic examination revealed a dermal granulomatous reaction with sarcoidal and tuberculoid granulomas (Figure 2a,b). Multiple asteroid bodies were observed in the cytoplasm of foreign-body-type multinucleated giant cells near translucent amorphous crystalline foreign bodies of various sizes (Figure 2c,d) that were birefringent on polarization (Figure 2e,f), consistent with silica crystals. A review of systems for pulmonary and other systemic symptoms was negative, and radiographs of the chest were unremarkable. Additionally, the patient had no risk factors for tuberculosis or other granulomatous infections. In the absence of systemic symptoms or findings suggestive of sarcoidosis or infectious causes, and given the presence of silica particles on histology, a final diagnosis of silica granuloma with asteroid bodies was made.

## 3. Discussion

Asteroid bodies are classically associated with sarcoidosis, but they are not at all pathognomonic, as our case highlights. The association of silica granulomas with asteroid bodies, to our knowledge, has not previously been reported, though it is not unexpected given the reports of asteroid bodies in several other types of foreign-body granulomas. Asteroid bodies have also been noted in various infectious and other granulomatous conditions (Table 1) [3,4]. 

The composition of asteroid bodies and the mechanisms of their formation remain a matter of ongoing investigation. Some existing evidence suggests they are products of microtubular disarray, collagen trapping, or ubiquitin-associated cytoskeletal abnormalities [5,6,7]. Others have postulated that the unique morphology of giant cell asteroid bodies may be attributable to hydrophobic properties of complex lipid constituents, which presumably accumulate within upon fusion of macrophages into giant cells [8,9].

Cutaneous silica granulomas have been sparsely described, though some have proposed they are underreported and underdiagnosed [2,10]. Despite the paucity of cases in the literature, silica is widely prevalent in the environment, and exposure can thus occur in a multitude of environments. Cutaneous silica granulomas often occur in individuals with a history of traumatic introduction of glass, sand, or other silica-containing particles into the skin. Silica implantation may occur even with minor trauma, often many years prior to cutaneous granuloma formation; as a result, exposure events may not be readily identified in the clinical history, as was the case in our patient [1,11,12]. The pathogenesis of cutaneous silica granuloma and its prolonged latency time remains elusive; theories include conversion to colloidal silica over time, eventually leading to a granulomatous reaction, or a type of delayed hypersensitivity response [11,13]. Given the wide discrepancy between the frequency of silica in the environment and the occurrence of silica granulomas, it is plausible that certain individuals are predisposed to this type of foreign-body reaction [11,14]. 

Silica foreign-body granulomas and sarcoidal granulomas can have near-identical histopathologic pictures, differentiated primarily by the presence of polarizable foreign bodies in the former. This finding was once generally understood to exclude a diagnosis of sarcoidosis, though more recent reports suggest that the boundaries may not be so clear. Several cases of systemic sarcoidosis have been reported in which polarizable foreign bodies are detected in cutaneous granulomatous lesion specimens [15,16,17]. Additionally, cases of cutaneous silica granuloma evolving into systemic sarcoidosis have been reported [18]. Our observation of asteroid bodies in silica granulomas adds to a histopathological picture that overlaps with sarcoidosis, highlighting the importance of clinico-pathological correlation. 

The optimal management of patients with cutaneous silica granulomas remains unclear. For limited cutaneous disease, excision is often performed with good effect, though recurrences are not uncommon [19]. Spontaneous resolution over several months has been noted in several cases [19]. 

While rare, the present case suggests that asteroid bodies can occur in silica foreign-body reactions in the absence of sarcoidosis. Clinicians and pathologists should be mindful of the overlapping presentation of foreign-body granulomas and sarcoidosis, which requires clinicopathological correlation. 

## Figures and Tables

**Table 1:** Various conditions in which asteroid bodies have been described.

Sarcoidosis	
Foreign-body granulomatous reactions	
	Silicone
	Beryllium
	Graphite
	Mercury alloy
	Polytef
Infectious disease	
	Tuberculosis
	Leprosy
	Histoplasmosis
	Sporotrichosis
	Cryptococcosis
	Aspergillosis
	Schistosomiasis
	Candidiasis
Annular elastolytic granuloma	
Necrobiotic xanthogranuloma	
Necrobiosis lipoidica	
Fibrocystic breast disease	
Tumors	
	Cystic teratoma
	Soft-tissue amyloidoma

## Data Availability

Data are contained within the article.
